# All Eyes on Me

**DOI:** 10.4269/ajtmh.20-0621

**Published:** 2020-10

**Authors:** Damasi F. Bayo, Nasreen S. Quadri

**Affiliations:** 1 Selian Lutheran Hospital, Arusha, Tanzania;; 2 University of Minnesota, Minneapolis, Minnesota

Normally, we start the day with morning prayer at the chapel inside the hospital compound. This day was like many others before it until at precisely 8:20 am when the emergency department nurse came directly to me looking for a representative from the pediatrics department. I looked around before quickly acknowledging I would be attending to this emergency myself. I ran toward the emergency department to find a 10-year-old boy convulsing continuously; his tonic-clonic motions mirroring my quickened breath from the adrenaline rush. My mind joined the quickened pace to calculate the loading dose of diazepam to intervene. My eyes surveyed his body from head to toe: semiconscious; drowsy; febrile; dyspneic; and multiple large blisters in the axilla, groin, back, and buttocks. A second type of calculations landed me at my diagnostic conclusion of suspected meningitis with secondary bacterial skin infections.

Once he stabilized, my eyes shifted to his father, a carpenter, who explained only a brief history of the events leading up to his presentation. I learned the boy was in standard six at a boarding school and had only returned home 7 days before as the country’s early response to the COVID-19 pandemic was school closures. His ailment began with a sudden onset of severe headache, gradual onset of high fever, progressive nausea, and vomiting that all spanned 1 day but must have felt like eternity to him.

I was elated to see my supervising pediatrician nearby. I reflected on the characteristics I admired about him: he is attentive to his patients with his time and also his financial resources when inevitably patients are unable to pay for the cost of investigative tests. Moreover, he embodies the genuine qualities of asking questions and teaching all levels of trainees at our hospital. Together, we reviewed the feasibility of a lumbar puncture, to draw out the cerebrospinal fluid of this young boy in search of answers, only to conclude the contraindications to this procedure with the blisters on his back.

Meningitis is a diagnosis we know far too well from our clinical experience in Tanzania. Worldwide mortality rates from meningitis are 20–40% in neonates, 5–10% in infancy, and childhood, with an astonishing 90% of cases occurring in children younger than 5 years. In Tanzania, the attack rate is 171 per 100,000. Once again, this young boy’s body was under attack by a disease process we knew all too well. I easily remembered a flood of information about meningitis as it was only a few weeks back that I was standing up in the chapel presenting pediatric meningitis as part of the continuing medical education lecture series. It was finally feeling as though all of these steps of education were amounting to an accumulation of knowledge that mattered.

In Tanzania, after graduating from 5 years of medical school and formal registration as a medical doctor, one must complete an internship at a regional referral, zonal, or national hospital in accordance with the Medical Council of Tanganyika. My internship consists of rotating through four major departments, including internal medicine, pediatric and child health, surgery and trauma, and obstetrics and gynecology with a sprinkling of experiences in other subspecialties such as ophthalmology, otolaryngology, and radiology. After 2 months on surgery, I landed in my current position as an intern doctor on the pediatrics team. I am fortunate to work with other intern doctors, a medical registrar, a supervising pediatrician, and a handful of visiting pediatricians. I’ve learned much about the experience and challenges of diagnosing and treating patients in a low-resource setting, which is the rule rather than the exception in our country. Day after day, I witness diseases of poverty such as malnutrition, HIV/AIDS, pneumonia, malaria, and diarrhea. We learn how to use what we have to save the lives of patients. In medical school, I was taught that 80% of the final diagnosis comes from thoroughly taking the history and physical examination, which I memorized right alongside all the other nuggets of knowledge. It is only now that I have come to fully realize the truth in that teaching as I see a lack of available investigations including magnetic resonance imaging or computerized tomography scan in our hospitals. These resources are only available at a few equipped private hospitals nearby, whereby most patients cannot afford to access them.

After 4 days of intravenous antibiotics aimed directly at treating his suspected meningitis, his relatives began demanding discharge from the hospital as he was outwardly appearing to have returned to his normal healthy state, despite the reality that his body was still fighting this potentially deadly pathogen if given the right circumstances. As I had been taught, I explained calmly the consequences of discharge before completing the prescribed 14 days of intravenous antibiotics for his condition and my worries about his clinical course. I could see in their eyes how much his father worried about attending to his job as it was the sole means of earning money for their family and how much the mother missed the rest of the family while attending to her sick son. I looked at the boy in front of me smiling, and it was as though he had not been ill and convulsing in front of my very eyes just days ago. Despite my best efforts, his family did not agree to stay, and we were forced to discharge him with the second best option we could offer of oral antibiotics for the remaining duration of therapy. I felt like a mechanic given the task of fitting the bus tires with nuts and bolts just as it was embarking on a journey carrying 30 passengers—and I chose the wrong bolt. However, I am realizing that patients and their families have autonomy in making these decisions, and frequently, these decisions are rooted in the heavy load of social and financial considerations impacting their everyday lives.

The next day, I again found myself in morning prayer at the chapel when this same young boy presented again with complaints of severe headache and low back pain. I felt like my heart was about to explode. The atmosphere in the chapel changed abruptly; it was a cold day during rainy season but suddenly I felt like I was close to a burning forest. All of the doctors in the chapel turned their eyes on me and my team questioning us simultaneously on how we could let this happen and why we had discharged the patient prematurely. It was like an antelope meeting a pride of lions in the wild, and there was nowhere else to run. At first, I remained silent, and the whole chapel was quiet like a pond without frogs. Though I tried to relay the conversations of the day before, only the pediatrics team could understand the depth of the story. Luckily for this young boy, following some further investigations and timely intravenous antibiotic therapy, he recovered completely.

This case and many others like it challenge me to gain a deeper understanding of Maasai society, where trust in traditional herbs trumps trust in hospitals and allopathic medicine. This results in late presentation of patients to hospitals in critical condition adding to the already existing challenges of treatment in low-resource settings. I have learned firsthand how these herbal therapies are not benign; they result in end-organ damage such as kidney failure and encephalopathy—an intoxication stemming from their very source of trust. There must be a way that we can share this reality with the community and change the trajectory of cases that present to our hospital day after day.

With each challenge I face, I gain a deep understanding of the complementary skills necessary to make a real difference in the lives of my patients. Thus far, I have learned that medical knowledge, cultural understanding, and political savvy are critical components to a holistic approach to community health care. Now I see that these skills are possessed by the most effective contributors to positive change. I desire to be among those ranks. Every day, I am trying to learn and adapt to different cultural norms while building my understanding of how to work in a complex environment with inadequate resources. It seems through this process that my childhood dream has come to fruition: to serve as a doctor driven by compassion for those in need of medical care, especially those with the least access.

